# Transrectal endoscopic drainage with vacuum-assisted therapy in patients with anastomotic leaks following rectal cancer resection

**DOI:** 10.1007/s00464-021-08359-4

**Published:** 2021-03-01

**Authors:** Mateusz Jagielski, Jacek Piątkowski, Grzegorz Jarczyk, Marek Jackowski

**Affiliations:** Department of General, Gastroenterological and Oncological Surgery, Collegium Medicum Nicolaus Copernicus University, 53-59 Św. Józefa St, 87-100, Toruń, Poland

**Keywords:** Anastomotic leak, Ileostomy, Rectal tumors, Vacuum-assisted therapy

## Abstract

**Background:**

Surgery is the gold standard for the treatment of malignant tumors of the rectum. Intestinal anastomotic leakage remains a serious complication of colorectal surgery. The efficacy and safety of transrectal endoscopic drainage by vacuum therapy in patients with intestinal anastomotic leakage after surgical treatment of middle and distal rectal tumors were assessed.

**Methods:**

Prospective analysis of treatment outcomes among patients undergoing surgery for middle and distal rectal tumors at the Department of General, Gastroenterological, and Oncological Surgery of the Ludwik Rydygier Collegium Medicum in Bydgoszcz and Nicolaus Copernicus University in Torun from 2016 to 2019 was conducted.

**Results:**

Seventy-nine patients with middle and distal rectal tumors underwent laparoscopic resection. Intestinal anastomotic leak was identified in 18 (22.79%) patients [all men, mean age 61.39 (43–86) years] during the postoperative period. Primary protective ileostomy was performed in 8/18 (44.44%) patients. All 18 patients were treated with endoluminal vacuum therapy via transrectal endoscopic drainage. The mean time from surgery to the diagnosis of leakage and initiation of endoscopic treatment was 16 (3–728) days. The mean number of endoscopic procedures per patient was 6 (1–11). The mean duration of endoscopic treatment was 22 (4–43) days. Complications of endotherapy occurred in 2/18 (11.11%) patients treated endoscopically for bleeding from the abscess cavity. Success of endoluminal vacuum therapy was achieved in 17/18 (94.44%) patients. Moreover, 5/18 (27.78%) patients required ileostomy during the endoscopic treatment. The mean follow-up period was 368 (118–724) days. Long-term success of transrectal endoscopic drainage using vacuum-assisted therapy was achieved in 15/18 (83.33%) patients.

**Conclusions:**

Endoscopic rectal drainage using vacuum-assisted therapy is an effective and safe minimally invasive treatment in patients with intestinal anastomotic leaks following resection procedures within the middle and distal rectum.

Surgical resection often combined with preoperative radiotherapy or radiochemotherapy is the standard treatment for patients with rectal cancer [1–3]. Despite neoadjuvant treatments, surgery remains the treatment of choice for rectal cancer [1–3]. Recently, many minimally invasive techniques have been proposed for abdominal surgery, including rectal surgery. Compared with conventional surgical treatment, minimally invasive techniques for the treatment of noninvasive rectal cancer, such as laparoscopic total mesorectal excision (LaTME) or transanal total mesorectal excision (TaTME), shorten the duration of hospitalization and improve short-term outcomes without affecting the outcomes of oncological treatment [4–6]. Moreover, minimally invasive access often facilitates the creation of a primary intestinal anastomosis without the need for stoma formation [4–7].

Despite the development of minimally invasive techniques and improved quality of perioperative care, the proportion of postoperative complications in colorectal surgery remains high [4–7]. In particular, anastomotic leak occurs in 6–22% of patients after colorectal resection and is associated with increased mortality in the postoperative period [8].

Intestinal anastomotic leak is defined as an interruption in the continuity of the anastomosis, resulting in spillage of the intestinal contents, which, depending on the location, may lead to the formation of an abscess or fecal peritonitis [9]. The therapeutic management of anastomotic leaks primarily depends on the patient’s clinical condition, which closely correlates with the location and extent of the leak [8, 9]. In the case of low intestinal anastomosis, as is the case with rectal surgery, spillage of intestinal contents through an anastomotic leak does not usually cause generalized peritonitis but causes local pelvic inflammation, resulting in pelvic abscesses [8, 9]. These patients are usually clinically stable, without symptoms of peritonitis [9].

In the case of minor anastomotic leaks, spontaneous closure of the defect is possible, but most patients require treatment [8, 9]. Clinically stable patients without the symptoms of peritonitis or sepsis may be managed conservatively, including nutritional support, broad-spectrum intravenous antibiotics, and observation, with interventional treatment reserved for cases of clinical deterioration or exacerbation of symptoms [8–10]. Radical surgical treatment consists of drainage, often combined with resection of a portion of the intestine and proximal colostomy, or abdominoperineal excision, resulting in the formation of a permanent stoma [10, 11]. Endoscopic treatment appears to be an intermediate method in the event of general clinical deterioration refractory to conservative management before a radical surgery is considered [12–14]. Nevertheless, constant development in advanced endoscopic techniques observed in recent years caused redefinition of endotherapy in the management of low anastomotic leaks. Currently, endoscopic vacuum therapy is considered primary management of low anastomotic leaks in stable patients without the symptoms of peritonitis or sepsis in experienced referral medical centers.

In our medical center, together with minimally invasive treatment of rectal cancer [7], we have introduced minimally invasive treatments for complications of colorectal surgery, such as endoscopic vacuum-assisted therapy. The present study assessed the efficacy and safety of transrectal endoscopic drainage with vacuum therapy in patients with anastomotic leaks after surgery for middle and distal rectal tumors.

## Materials and methods

The study was approved by the Ethics Committee of our medical center and our university. All patients gave their informed consent for the procedures.

Prospective analysis of the outcomes of all consecutive patients undergoing surgical treatment for middle and distal rectal tumors at the Department of General, Gastroenterological, and Oncological Surgery of the Ludwik Rydygier Collegium Medicum in Bydgoszcz and Nicolaus Copernicus University in Torun from 2016 to 2019 was conducted.

All patients were referred for surgery following oncological team consultation. Some patients received neoadjuvant therapy prior to surgery. All patients had performed total mesorectal excision (TME). If the tumor was located ≤ 5 cm from the pectinate line, patients were qualified for TaTME. TaTME was also performed if the tumor was located 5–10 cm from the pectinate line and appropriate anatomical conditions were present (obesity, male sex, narrow pelvis). In all other cases, patients with tumors of the middle rectum underwent LaTME. Intraoperative revision of the quality of intestinal anastomosis was performed during each surgical procedure. Primary protective ileostomy was performed during surgery (LaTME or TaTME) in part of patients. Indications for primary protective ileostomy were: technical difficulties during resection procedure mainly related to previous neoadjuvant treatment or intestinal anastomosis performed in distance smaller than 50 mm from the anal verge.

If blood tests indicated elevated inflammatory markers or if severe clinical symptoms suggestive of anastomotic dehiscence appeared during the postoperative period, endoscopic examination of the lower gastrointestinal tract was performed to assess the integrity of the anastomosis. If signs of an anastomotic leak were found, contrast multiphase computed tomography of the abdomen and pelvis was performed for definitive diagnosis. Clinically stable patients with confirmed intestinal anastomotic leak and without the signs of diffuse peritonitis or sepsis were referred for transrectal endoscopic drainage.

**Fig. 1:**
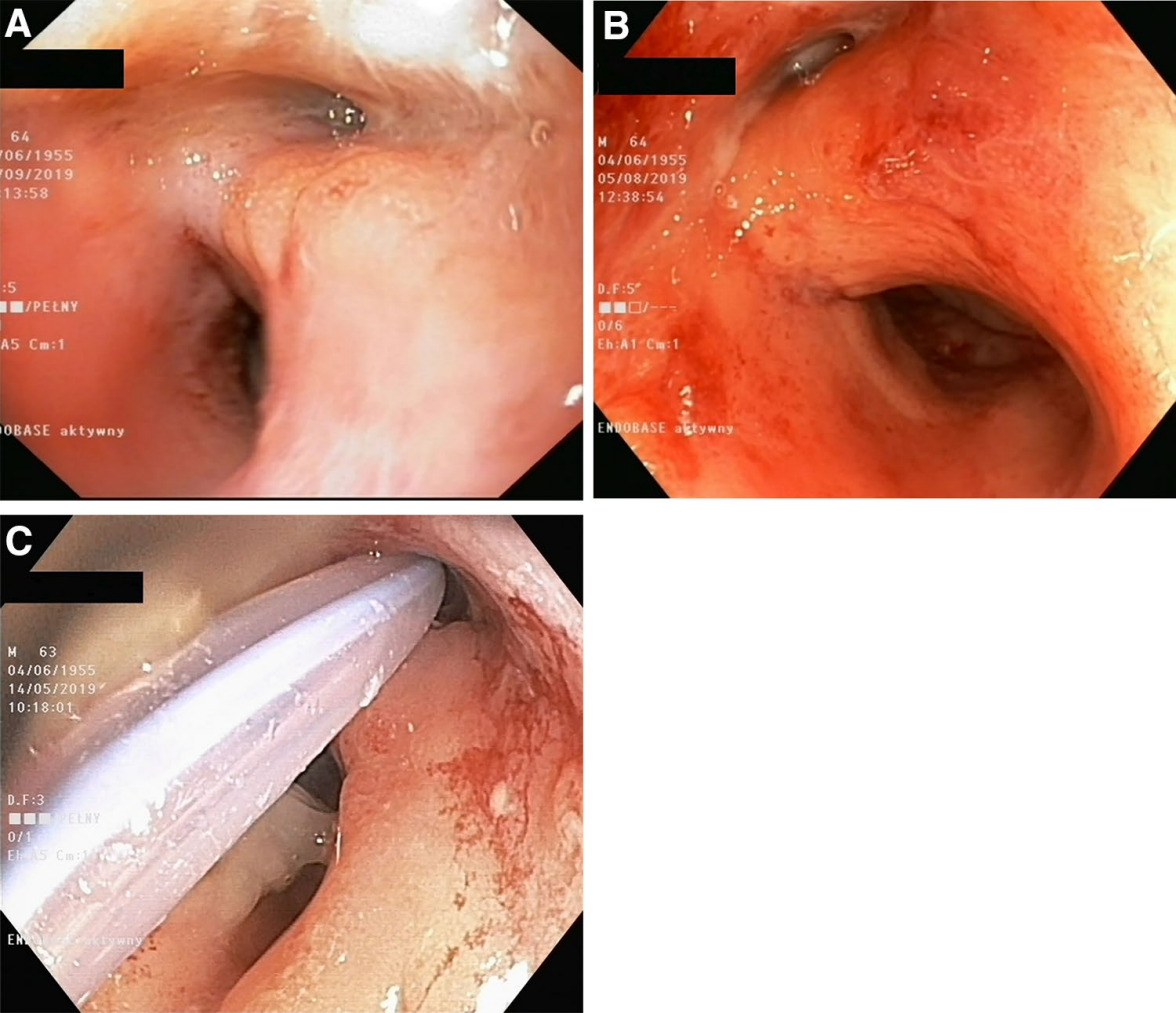
**A**–**C** The anastomotic leak did not exceed 30 mm on endoscopic examination (**A**, **B**). An 8-Fr drainage catheter was introduced transrectally under endoscopic and fluoroscopic guidance through the site of the leak (**C**)

If the anastomotic leak did not exceed 30 mm on endoscopic examination, an 8-Fr drainage catheter was introduced transrectally under endoscopic and fluoroscopic guidance through the site of the leak (Fig. 1). Its distal end was left within the abscess cavity for flushing, which usually consisted of 50 mL of saline every 6 h after the procedure. If further progression of anastomotic dehiscence was noted during subsequent endoscopies and exceeded 30 mm, then transrectal vacuum-assisted therapy was administered.

**Fig. 2:**
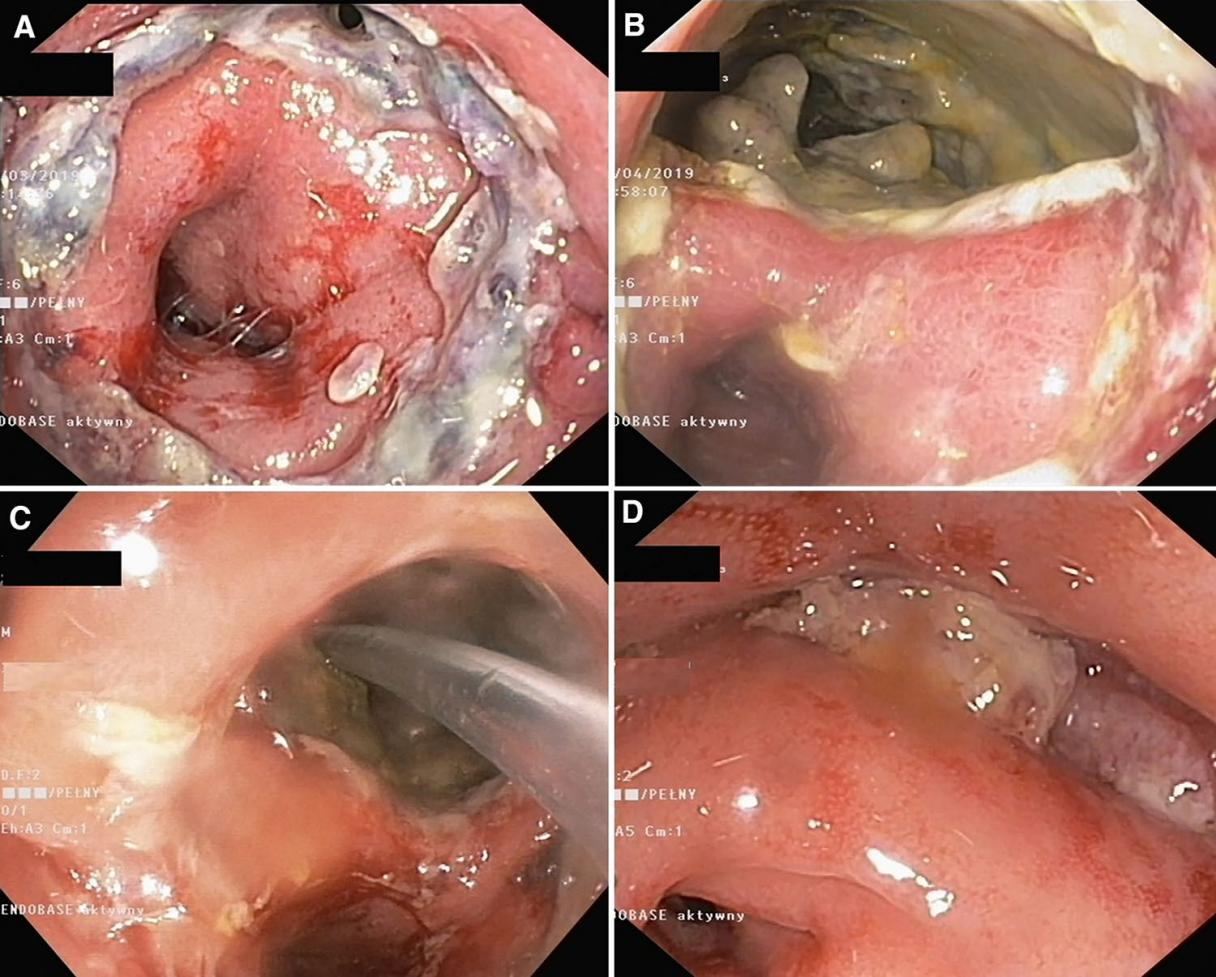
**A**–**D** Intra-abdominal vacuum-assisted therapy using the transrectal endo-SPONGE drainage system. Patient on Day 3 after TaTME. Initially, a small intestinal anastomotic leak noted in endoscopy (**A**) drainage catheter was introduced transrectally into the abscess cavity through the site of the leakage under endoscopic and fluoroscopic control. Progression of anastomotic dehiscence was found after 7 days of drainage during the following endoscopic procedure (**B**). The patient underwent transrectal vacuum therapy (**C**), which continued for 20 days. A follow-up endoscopic study performed at 3 months demonstrated closure of the leakage with granulomatous tissue (**D**)

**Fig. 3:**
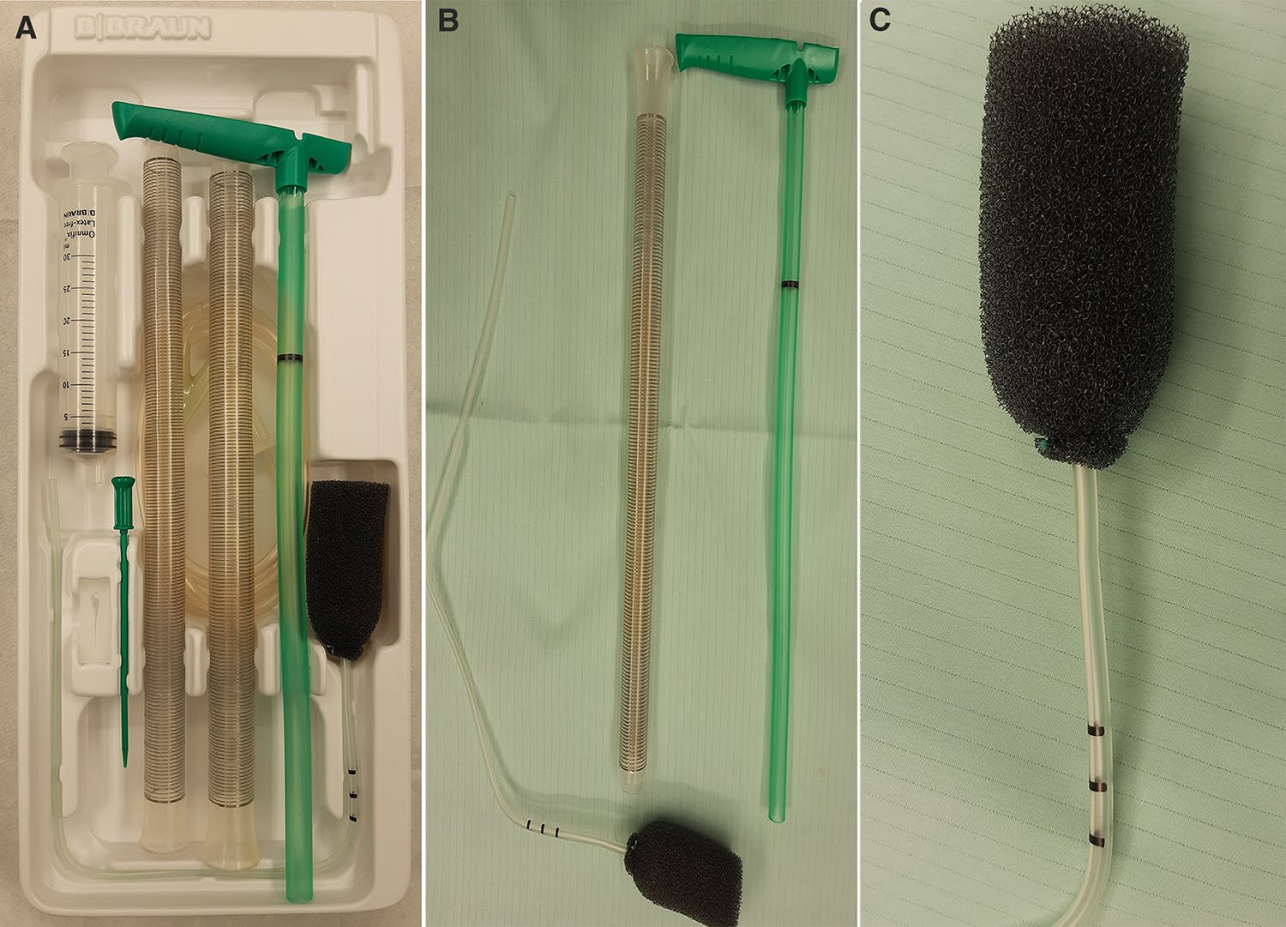
**A**–**C** The commercially available endoluminal vacuum system (Endosponge®, **B** Braun, Melsungen, Germany) for treatment of patients with anastomotic leaks

In cases where the magnitude of the leak exceeded 30 mm during the first or subsequent endoscopic examinations, transrectal vacuum-assisted therapy was implemented using transrectal endoscopic drainage (Fig. 2) with Endo-SPONGE (B. Braun Medical B.V., Melsungen, Germany) set (Fig. 3). Depending on the size of the anastomotic leak and the abscess, a sponge with a Redon drain included in the pack was cut to size, or more than one sponge was used in cases of large areas of dehiscence exceeding half of the anastomosis circumference. After endoscopy, the drain was connected to a subatmospheric pressure system, with a vacuum pressure of 80–130 mm Hg. Endo-SPONE kits were changed during subsequent endoscopies, which was repeated every 3–5 days. During the following endoscopic procedure, after removing the old set, the abscess cavity was rinsed repeatedly with a disinfectant solution (Granudacyn). If tissue fragments adhering to the abscess wall were present within the abscess, direct endoscopic necrosectomy was performed before introducing a new set. An endoscope was introduced into the abscess through the leakage site, and fragments of necrotic tissue were removed using a Dormia basket or polypectomy loop under endoscopic guidance. Then, after draining the contents of the abscess cavity, a new drainage kit with a vacuum dressing was introduced at the end of the procedure. If no endoscopic regression of lesions was observed during the three consecutive endoscopic procedures involving the replacement of the Endo-SPONGE dressing, the patients underwent protective ileostomy.

All endoscopic procedures were performed under sedation with propofol with anesthetic supervision. Endoscopic procedures were performed using flexible videocolonoscopes (Olympus CF-Q165L).

Endoluminal vacuum therapy using transrectal endoscopic drainage continued until a successful outcome was achieved, which was defined as the resolution of clinical signs and complete resolution of an abscess with leak closure by granular tissue or as the resolution of clinical signs and reduction in the size of the abscess to a diameter below 30 mm (confirmed by imaging) with filling of an abscess with granulation tissue (confirmed by endoscopy). If a successful outcome was not achieved within 50 days of endoscopic drainage or after 10 endoscopic procedures or if the clinical deterioration was observed during endoscopic drainage, the patient was referred for radical surgical treatment. Surgery was performed during endoscopic treatment, and endoscopic drainage was continued even after radical surgical treatment.

Patients were followed up after successful transrectal endoscopic drainage with vacuum-assisted therapy. During follow-up, endoscopic examinations of the lower gastrointestinal tract were repeated every 1, 3, 6, 9, 12, 18, and 24 months. During the observation period, imaging studies (contrast-enhanced computed tomography of abdomen and pelvis) were also performed at 6, 12, and 24 months. If a relapse of an abscess with intestinal fistula in the area of anastomosis was found, the endoscopic treatment was repeated. Moreover, during follow-up, patients with ileostomy were referred for a procedure to restore continuity of the gastrointestinal tract after anastomotic leak closure.

## Definitions

Complications of endotherapy were defined as adverse events occurred during endoscopic drainage.

Success of endoluminal vacuum therapy using transrectal endoscopic drainage was defined as the resolution of clinical signs and complete resolution of an abscess with leak closure by granular tissue or as the resolution of clinical signs and reduction in the size of the abscess to a diameter below 30 mm (confirmed by imaging) with filling of an abscess with granulation tissue (confirmed by endoscopy), which allowed endotherapy to be completed.

Recurrence of the pelvic abscess was determined as the collection size > 30 mm or relapse of clinical symptoms during a follow-up.

Long-term success of transrectal endoscopic drainage using vacuum-assisted therapy was defined as lack of recurrence of symptoms, lack of abscess recurrence, and absence of intestinal fistula in the area of the anastomosis.

## Statistical analysis

All statistical calculations were performed using STATISTICA software v.10.0 (StatSoft). Quantitative variables are described as arithmetic means, standard deviations, minimum and maximum values (range), and 95% confidence intervals (CIs). Qualitative variables are presented using numbers and percentages (proportions).

## Results

Seventy-nine patients with middle and distal rectum cancer underwent laparoscopic resection at our center from 2016 to 2019. Neoadjuvant therapy—radiotherapy or radiochemotherapy—was used in 73/79 (92.41%) patients before surgery, but most of them (62/79 [78.48%] patients) had short course neoadjuvant treatment. Qualification for oncological treatment was determined by oncology specialists who are a part of our team. The mean time from radiotherapy to surgery was 10.57 (5–56) days. Overall, 38/79 (48.10%) patients underwent TaTME and 41/79 (51.90%) patients underwent LaTME.

**Fig. 4:**
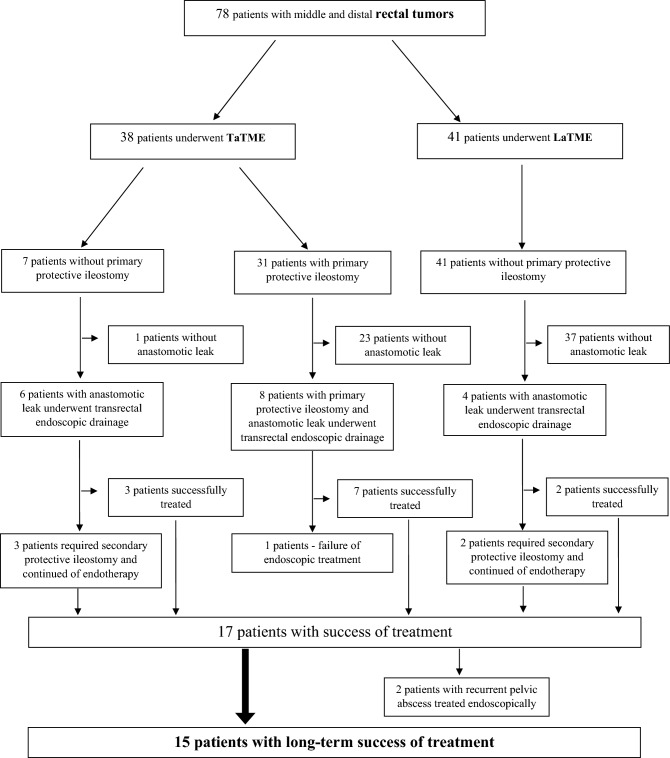
The consort flowchart of patients selection

In 18/79 (22.79%) patients [all men, mean age 61.39 (43–86) years] (Table 1), a leak was diagnosed in the postoperative period, and 8/18 (44.44%) patients underwent primary protective ileostomy (Fig. 4). The average distance form the dentate line to the location of leaks in endoscopic view was 40 [20–100] mm. In 16/18 (88.89%) patients, intestinal anastomotic leakage exceeded 30 mm on endoscopic examination. All 16 patients underwent endoluminal vacuum-assisted therapy using transrectal endoscopic drainage. In the remaining 2/18 (11.11%) patients, the size of the anastomotic leak did not exceed 30 mm in the endoscopic examination. In these patients, an 8-Fr drainage catheter was introduced through the site of the leak into the abscess cavity to enable wash-out in the postoperative period. In both patients, anastomosis dehiscence of over 30 mm was found during the second endoscopic procedure on Day 8 of endoscopic drainage, and they were referred for drainage with endoluminal vacuum-assisted therapy.

**Table 1:** Detailed characteristics of patients treated with endoluminal vacuum therapy via transrectal drainage

	All (*n* = 18)
Age, mean, [range]	61.39 [43–86]
Gender, n male (%)	18 (100%)
BMI (kg/m2), mean, [range]	23.86 [17.2–31.1]
ASA (class), *n*, (%)	
I	2 (11.11%)
II	11 (61.11%)
III	4 (22.22%)
IV	1 (5.56%)
Tumor size (mm), mean, [range]	48 [15–72]
Depth of invasion (grade), *n*, (%)	
T1	2 (11.11%)
T2	4 (22.22%)
T3	8 (44.45%)
T4	4 (22.22%)
Pathological stage, *n*, (%)	
G1	3 (16.67%)
G2	11 (61.11%)
G3	4 (22.22%)
Neoadjuvant chemoradiotherapy, *n*, (%)	16/18 (88.88%)
Primary protective ileostomy, *n*, (%)	8/18 (44.44%)
Distance form dentate line to location of leak (mm), mean, [range]	40 [20–100]

*BMI* body mass index, *ASA* American Society of Anesthesiology

The mean duration from surgery to the diagnosis of leakage and initiation of endoscopic therapy was 16 (3–728) days. The average number of endoscopic procedures with vacuum dressing replacement was 6 (1–11). The average duration of endoscopic treatment was 22 (4–43) days. Complications of endotherapy occurred in 2/18 (11.11%) patients: they were diagnosed with bleeding from the abscess cavity. Bleeding was controlled endoscopically by the injection of hemostatic powder (Hemospray) into the abscess cavity, and transfusions of red blood cell concentrate and freshly frozen plasma were needed during the endotherapy.

All patients undergoing endotherapy of intestinal leaks were on an oral diet, and additional parenteral supplementation was necessary in 11/18 (61.11%) patients. Moreover, 8/18 (44.44%) patients required broad-spectrum intravenous antibiotics during endoscopic drainage. In 5/18 (27.78%) patients, ileostomy construction was necessary due to the lack of endoscopic regression of lesions during the subsequent three procedures using vacuum dressing replacement.

Furthermore, 1/18 (5.56%) patient required radical surgical treatment because endoscopic drainage was ineffective. On Day 52 of endoluminal vacuum-assisted therapy using transrectal endoscopic drainage, a fragment of the intestine was resected along with the leaking anastomosis together with pelvic abscess drainage and construction of a permanent colostomy. Following surgery, endoscopic drainage with a vacuum dressing within the remaining rectal stump was conducted for the next 11 days.

Endoscopic leak treatment using vacuum-assisted therapy was successful in 17/18 (94.44%) patients (success of endoluminal vacuum therapy using transrectal endoscopic drainage). The mean follow-up period was 368 (118–724) days.

During the follow-up period, two patients had a recurrence of the pelvic abscess with persistent intestinal fistula within the anastomosis. They again underwent endotherapy with vacuum-assisted therapy and transrectal drainage. The abscess cavity was accessed through the intestinal fistula. In these patients, the duration of endoscopic treatment was much shorter (15 days) with the need for four endoscopic procedures. Thus, 17/18 (94.44%) patients with a low intestinal anastomotic leak avoided radical surgical treatment.

In all 13 patients with protective ileostomy, surgical reconstruction of continuity of the gastrointestinal tract with closure of ileostomy was performed average of 14 (11–25) weeks form the end of endotherapy.

Successful long-term outcomes of endoscopic treatment were found in 15/18 (83.33%) patients (long-term success of transrectal endoscopic drainage using vacuum-assisted therapy).

## Discussion

This study introduces some novelties to the state of knowledge in order to improve endoscopic treatment’s results in patients with anastomotic leaks following rectal cancer resection. In the study, innovatory treatment approach based on the diameter of anastomotic leak and its endoscopic image was presented, which is strictly connected with time from surgery. This management strategy differs significantly from earlier approaches and is related to high efficacy.

In clinically stable patients with anastomotic leak following resection procedure in the rectum who do not have symptoms of peritonitis or sepsis, conservative management with watchful waiting is possible, and interventional treatment can be reserved for cases of clinical deterioration or symptom exacerbation. Radical surgical treatment involves surgical procedures, which often results in the creation of a permanent stoma [10, 11]. Endoscopy with transrectal vacuum-assisted drainage appears to be the intermediate treatment in patients with intestinal anastomotic leak before radical surgery whose general condition is not improving with conservative treatment [9, 12–14]. The advantage of this minimally invasive treatment method over other drainage techniques lies in the possibility of removing the purulent contents by the transrectal route and reversing the pressure gradient through suction, which prevents the persistence of anastomotic leaks or intestinal fistulas. A properly inserted transrectal suction drainage allows for local control of pelvic infection, thus preventing diffuse peritonitis or sepsis. In addition, filling the cavity with a suction sponge reduces the dehiscence and potentially prevents the constant communication of intestinal contents with the abscess cavity. The major indisputable advantage of this method is associated with the role of the vacuum, which promotes anastomotic healing by augmenting vascular perfusion and increasing the probability of granulation tissue formation [15, 16].

To begin with, we will present the main novelties of this study, which will be described more thoroughly in the following paragraphs. First of all, we introduced completely different approach in case of minor anastomotic leaks (< 30 mm). The novelty of our strategy is about introduction of drainage catheter transrectally through the site of the leak into abscess cavity for flushing with no need for dilatation of anastomotic leak necessary for implementation of Endo-SPONGE. Secondly, we showed how important optimal timing of endotherapy and frequency of Endo-SPONGE replacement are. The management algorithm with timing according to our indications is optimal due to high efficacy of endoscopic treatment. Thirdly, opposing to other studies, we described approach in which it was essential to know when to complete endotherapy—not necessarily at the moment of complete closure of the anastomotic defect, which could take months, but when the abscess cavity involuted to at least 30 mm and appropriate healing potential was achieved through epithelialization of the abscess walls with granulation tissue. The approach presented above shortens time of endotherapy without having effect on efficacy. Fourthly, we showed that protective ileostomy increases results of endoscopic treatment and shortens time of endotherapy. Although we showed that in part of patients without protective ileostomy, effective endoscopic treatment of anastomotic leaks is also possible, but in these cases, time of endotherapy may be longer.

In our study, endoscopic treatment using transrectal vacuum-assisted drainage was successful in 17/18 (94.44%) patients. The mean time of endotherapy was 22 days, with an average of six procedures per patient. In the first description of the use of endoluminal vacuum therapy for the treatment of intestinal anastomotic leak, Weidenhagen et al. achieved therapeutic success in 28/29 (96.55%) patients, with the mean duration of endoscopic treatment of 34 days and a mean of 11 endoscopic procedures per patient [12]. van Koperen et al. used the endovacuum therapy to treat low intestinal leaks and achieved therapeutic success in9/16 (56.25%) patients [14]. However, as the authors indicated, poorer treatment outcomes than those observed by Weidenhagen et al. [12] were due to a longer time from surgery to the start of endoscopic treatment of anastomotic leaks [14]. Similarly, in our study, the longer period from surgery to the start of endotherapy did not so much affect the therapeutic success itself but extended the time of endoscopic treatment, increased the number of endoscopic procedures, and increased the number of relapses. In our opinion, this was caused by fibrosis within the anastomosis, which occurs overtime. Progressive fibrosis causes a small anastomotic leak to develop into a well-defined intestinal fistula, and a persistent leak leads to the formation of a pelvic abscess that communicates through the fistula with the lumen of the gastrointestinal tract. Over time, the walls of the abscess mature and become fibrosed. These changes reduce the effectiveness of endotherapy, extending treatment time by increasing the number of endoscopic procedures and poorer long-term results, which is associated with a more frequent recurrence of abscesses. On the other hand, as observed in our study, early endoscopic drainage interventions in cases of minor leaks can lead to an increase in leak circumference, which also prolongs the time of endotherapy, due to the prolonged time required for a large defect to heal. Thus, it is difficult to determine the optimal timing for the start of endoscopic treatment in patients with a low intestinal anastomotic leak.

The time from surgery to the beginning of transrectal vacuum-assisted drainage plays a critical role in the effectiveness of endotherapy. As shown above, too early endoscopic intervention may result in increased size of an intestinal anastomosis defect, whereas late initiation of endotherapy for anastomotic leakage is associated with a high risk of recurrence of pelvic abscesses [14, 17]. Both situations increase the duration of endoscopic treatment and reduce the effectiveness of endotherapy.

In a study by Weidenhagen et al., 24/29 (82.76%) patients underwent protective stoma formation [12]. In our study, 8/18 (44.44%) patients had a primary protection ileostomy, another 5/18 (27.78%) patients required ileostomy during endotherapy, which resulted in effective endoscopic therapy. A decompressing stoma plays a vital role in the endoscopic treatment using transrectal vacuum-assisted drainage. Draining the intestinal contents through the stoma prevents their contact with the anastomosis, which further promotes healing of tissues within the dehiscence and improves the effectiveness of endotherapy. Moreover, protective ileostomy itself, especially performed during TaTME, reduces the number of complications, mainly anastomotic leaks [7]. This observation prompted us to create protective ileostomy during each TaTME procedure in our facility, which improved short- and long-term treatment outcomes in these patients.

What is important, the size of the anastomotic defect is of no clinical significance, but the size of abscess cavity behind it is crucial. In our opinion, there is no need for uncontrolled dilatation of anastomotic defect in order to achieve wide access to the abscess cavity, which makes introduction of Endo-SPONGE set possible. In our medical center, if the anastomotic leak does not exceed 30 mm on endoscopic examination, an 8-Fr drainage catheter is introduced transrectally under endoscopic and fluoroscopic guidance through the site of the leak. Its distal end is left within the abscess cavity for flushing, which also allows us to control the infection and to observe the patient during drainage. The active drainage period makes spontaneous demarcation and limitation of anastomotic leak or complete regression of abscess possible under controlled infection, which results in positive clinical effects. If further spontaneous progression of anastomotic dehiscence is noted during subsequent endoscopies and exceeded 30 mm, then transrectal vacuum-assisted therapy is administered without need for dilatation of anastomotic defect.

Finally, we would like to provide some technical details related to the endoscopic transrectal vacuum-assisted drainage. The basis of transrectal endotherapy using vacuum dressings is repeated endoscopic treatment with wash-out of the abscess cavity, accessed through the site of anastomosis. Thorough and repeated endoscopic treatments during drainage increase the effectiveness of therapy. The intervals between revision endoscopic procedures with possible endoscopic necrosectomy should not, in our opinion, be longer than 5 days, to prevent the growth of granulation tissue from the healing abscess into the sponge. Treatments at intervals longer than 5 days thus increase the risk of complications such as bleeding. It is also essential to know when to complete endotherapy—not necessarily at the moment of complete closure of the anastomotic defect, which can take months, but when the abscess cavity involutes to at least 30 mm and appropriate healing potential is achieved through epithelialization of the abscess walls with granulation tissue. If the abscess cavity is residual and fully coated with granulation tissue, then spontaneous closure of the anastomotic defect will occur without the need for further intervention.

In our study, the commercially available endoluminal vacuum system (Endosponge®, B. Braun, Melsungen, Germany) (Fig. 3) was used in treatment of patients with anastomotic leaks following rectal cancer resection. The described Endo-SPONGE system is not available in some countries. In some publications, the authors describe efficacy of homemade devices in the endoscopic treatment of anastomotic leaks after surgery [18–20]. We never used that kind of devices due to availability of Endo-SPONGE system. Although basing on data from literature, homemade endoscopic system vacuum-assisted devices may be effectively used in treatment of patients with anastomotic leaks following rectal cancer resection in countries, where Endo-SPONGE is not available.

The main limitations of the study included a lack of randomization and the fact that the study was conducted in a selected group of patients from a single center. Another limitation of this study is male only cohort. Most current knowledge regarding diagnostic and therapeutic management of patients with intestinal anastomotic leaks in colorectal surgery comes from studies, such as those shown above, which supports the need for and validity of such publications.

No clear guidelines exist for the management of intestinal anastomotic leaks in colorectal surgery, highlighting the need for further research regarding the therapeutic management of these patients. Our results of effective treatment of intestinal leaks using endoscopic vacuum-assisted therapy, together with the absence of serious complications, suggest that with careful selection of patients, endotherapy may be the appropriate therapeutic option for treating gastrointestinal anastomotic leaks and preventing radical surgical interventions.
